# Recall of facial expressions and simple orientations reveals competition for resources at multiple levels of the visual hierarchy

**DOI:** 10.1167/19.3.8

**Published:** 2019-03-21

**Authors:** Viljami R. Salmela, Kaisu Ölander, Ilkka Muukkonen, Paul M. Bays

**Affiliations:** viljami.salmela@helsinki.fi; kaisu.olander@helsinki.fi; ilkka.muukkonen@helsinki.fi; pmb20@cam.ac.uk; Department of Psychology and Logopedics, University of Helsinki, Helsinki, Finland; Department of Psychology, University of Cambridge, Cambridge, UK; Department of Psychology and Logopedics, University of Helsinki, Helsinki, Finland; Department of Psychology and Logopedics, University of Helsinki, Helsinki, Finland; Department of Psychology, University of Cambridge, Cambridge, UK

**Keywords:** *working memory*, *precision*, *population coding*, *face*, *expression*, *emotion*, *orientation*

## Abstract

Many studies of visual working memory have tested humans' ability to reproduce primary visual features of simple objects, such as the orientation of a grating or the hue of a color patch, following a delay. A consistent finding of such studies is that precision of responses declines as the number of items in memory increases. Here we compared visual working memory for primary features and high-level objects. We presented participants with memory arrays consisting of oriented gratings, facial expressions, or a mixture of both. Precision of reproduction for all facial expressions declined steadily as the memory load was increased from one to five faces. For primary features, this decline and the specific distributions of error observed, have been parsimoniously explained in terms of neural population codes. We adapted the population coding model for circular variables to the non-circular and bounded parameter space used for expression estimation. Total population activity was held constant according to the principle of normalization and the intensity of expression was decoded by drawing samples from the Bayesian posterior distribution. The model fit the data well, showing that principles of population coding can be applied to model memory representations at multiple levels of the visual hierarchy. When both gratings and faces had to be remembered, an asymmetry was observed. Increasing the number of faces decreased precision of orientation recall, but increasing the number of gratings did not affect recall of expression, suggesting that memorizing faces involves the automatic encoding of low-level features, in addition to higher-level expression information.

## Introduction

Visual working memory retains information relevant to our current cognitive task, even when that information is no longer visible. Working memory is resource-limited, however, so only a few items can be remembered precisely. The precision of working memory representations has mainly been studied for primary visual features, using continuous recall tasks. In these tasks, one of several items in memory is cued, and participants adjust a probe stimulus to match the relevant feature—memory precision is quantified based on the dispersion of adjustment errors. A decline in recall precision with increasing memory load has been shown for color (Bays, Catalao, & Husain, 2009; van den Berg, Shin, Chou, George, & Ma, 2012; Wilken & Ma, 2004; Zhang & Luck, 2008), orientation (Bays & Husain, 2008; Salmela & Saarinen, 2013; van den Berg et al., 2012; Wilken & Ma, 2004), length (Palmer, 1990), location (Bays & Husain, 2008; Schneegans & Bays, 2016), spatial frequency (Wilken & Ma, 2004), and simple contours (Salmela, Lähde, & Saarinen, 2012; Zhang & Luck, 2008). In this study we present observers with images of human faces with continuously varying emotional expressions to study the precision of memory for more complex and naturalistic representations.

In comparison to primary visual features, faces are perceptually much richer. It has been suggested that the capacity of visual working memory is smaller for perceptually complex than simple objects (Alvarez & Cavanagh, 2004). However, with increasing encoding duration the difference between simple and complex or real world objects decreases (Brady, Stormer, & Alvarez, 2016; Eng, Chen, & Jiang, 2005). With long encoding duration, there is a recall advantage for upright faces compared to other complex objects and inverted faces (Curby & Gauthier, 2007), which could be a consequence of a unique facial memory system, or simply that perceptual expertise and experience enhance memory performance (Curby, Glazek, & Gauthier, 2009). In a study that attempted to control for memory–test item similarity, an advantage was found for storing facial identities over simple line orientations (Jiang, Shim, & Makovski, 2008). A memory advantage for faces is consistent with their importance in social interaction and communication, and evidence for specialized neural mechanisms for processing facial information (Tsao & Livingstone, 2008). A study using reproduction from a continuous face space found that precision was better for upright than inverted faces when studied along with varying gender and age (Lorenc, Pratte, Angeloni, & Tong, 2014). Some studies also suggest different memory performance for different expressions (e.g., better recall for faces with angry than happy expressions; Jackson, Linden, & Raymond, 2014). For recognition of facial expressions, in contrast, several studies have found an advantage for happy expressions (Leppanen & Hietanen, 2004; Svard, Wiens, & Fischer, 2012).

Most of the previous studies listed above have investigated memory for faces in terms of a fixed memory capacity or probability of correct recall. Here, we investigated memory precision and the effect of memory load for different facial expressions using a reproduction design. Our stimuli were face images morphed between neutral expressions and happy, angry, fearful, disgusted, or sad expressions. After a memory delay, observers adjusted a probe face to match the expression of the face in a cued spatial location, and the distribution of adjustment errors was used as a measure of memory precision. We compared these results to an experiment testing recall of oriented gratings. Finally, we investigated competition for memory resources between high- and low-level objects, in a task in which observers were required to simultaneously memorize the expressions on a face and the orientations of gratings.

For low-level features, the effect of set size on distributions of recall errors has been related to the underlying neural representations via a neural resource model (Bays, 2014; Schneegans & Bays, 2017). This model explains the decline in memory precision with set size as a decrease in the signal-to-noise ratio in neural populations tuned to the relevant stimulus feature. Previous implementations of this model have been for primary visual features that are naturally parameterized in a circular space (e.g., orientations). Here we modified the neural resource model to generate responses within a bounded one-dimensional space, allowing us to fit the model to data from both orientation and facial expression reproduction tasks.

## Materials and methods

### Participants

In total, 14 healthy volunteers (6 men, 8 women, aged 21–34) with normal or corrected-to-normal vision participated in the experiments. All the experiments were conducted in accordance with the Declaration of Helsinki. The participants gave written informed consent and the experiments were approved by the Ethics Review Board in the Humanities and Social and Behavioural Sciences of the University of Helsinki. The volunteers each participated in two or three of Experiments 1A–1C, with each experiment conducted on a different day. In total, data from nine participants was collected for each experiment.

### Stimuli

The stimuli consisted of images of faces differing in emotional intensity, and Gabor gratings differing in orientation. The spatial frequency of the gratings was 1.3 c/° of visual angle and they were windowed by 2D Gaussian function with a standard deviation of 0.77°. Face images were generated based on 60 identities, each with five emotional expressions (angry, disgusted, fearful, happy, and sad) and one neutral expression, drawn from the Faces (Ebner, Riediger, & Lindenberger, 2010) and Radboud (Langner et al., 2010) databases. Abrosoft FantaMorph software was used, separately for each identity and emotion, to create a set of 100 morphed images in which the first image depicted the neutral face (0% emotional intensity) and last image the full emotional expression (100% emotional intensity). With MATLAB (MathWorks, Natick, MA), the images were converted to grayscale, scaled to a uniform size, and cropped with an oval mask. The mask outline was a Gaussian two-component radial frequency (RF) pattern resembling a face shape (radius of the base circle: 2.5°; Component 1: RF = 2, amplitude: 0.55°, phase = 270°; Component 2: RF = 3, amplitude: 0.10°, phase = 180°). The RMS contrast and the mean luminance of the images were set to 0.2 and 145 cd/m^2^, respectively. The width and height of each face were 4 and 5.8 degrees of visual angle, respectively.

### Equipment

The stimuli were presented on a VIEWPixx display (VPixx Technologies Inc., Saint-Bruno-de-Montarville, QC, Canada) at 100 Hz refresh rate. Participants sat at 92 cm distance from the display and the viewing area subtended 29.5° × 18.8° (display resolution 1920 × 1200 pixels). Participants' head was placed in a chin and forehead support. Experiments were conducted in a dimly lit room. The participants' eye movements were tracked with an EyeLink 1000 (SR Research Ltd., Ottawa, ON, Canada) eye-tracker.

### Procedure

#### Experiment 1A

**Figure 1:**
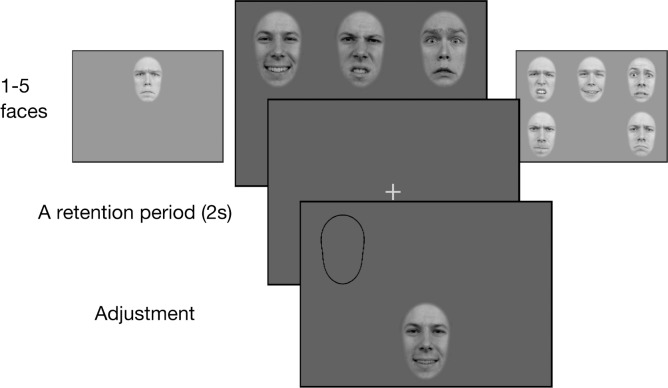
Experiment testing memory for faces expressing different emotions (Experiment 1A). A display containing between one and five faces was presented (duration 1 s/face), followed, after a 2 s retention interval, by a spatial cue and probe face. The participant adjusted the emotional intensity of the probe face to match the face that had appeared at the cued location (the target face). Emotion and identity of the probe always matched the target. The face images in the Figure 1 are from Radboud Faces Database (http://www.socsci.ru.nl:8180/RaFD2/RaFD); all of their images can be used in strictly scientific publications.

Recall was tested with a method of adjustment. On each trial, participants first viewed a display containing between one and five faces, each of which had a different identity, emotional expression, and intensity of expression. The display duration was 1 s per face, (e.g., 4 s for a four-face display). After a 2 s blank retention period, a probe face was displayed and the participant adjusted, by pressing left and right arrows of the keyboard, the intensity of the probe to match the intensity of the target face cued with an outline shape at its previous location (Figure 1). The identity and emotional expression of the probe and target faces were always the same. Feedback was given in the form of a beep sound if the adjustment error was greater than 25%. The initial intensity of the probe face was chosen randomly.

Each set size was tested in a separate block, and blocks were ordered in one of two ways. The first group (four participants) was tested with set sizes in order: 1, 2, 4, 3, 5, 1; the second group (five participants) in order: 1, 3, 5, 2, 4, 1. The rationale was to control for and assess possible learning effects, since overall the testing took several hours. Each participant started with set size one in order to practice the task. The overall learning effect was measured by repeating the set size one condition at the end of testing. Learning effects could be assessed at the level of set sizes by comparing the two participant groups (e.g., for three faces, Group 1 had more previous practice on the memory task than Group 2).

Each of the first five blocks consisted of 600 trials. The identities, emotions, and intensities were balanced within a block so that each identity was probed 10 times, twice with each of the five emotions, once with intensity less than 50%, and once with intensity greater than 50%. Ten different intensity levels were used ranging uniformly from 10*%* to 91*%*. The final set size one block consisted of 300 trials in which each identity–emotion combination was tested once.

Eye movements were recorded throughout the experiment, but participants were not given any specific instructions about where to look.

#### Experiment 1B

To compare memory precision for faces and primary features, Experiment 1B replaced the face stimuli with randomly oriented Gabor gratings, tested at set sizes of 1, 3, and 5. Participants adjusted the orientation of a probe grating to match the remembered orientation of a grating indicated by a cue. The experiment was identical to Experiment 1A in all other respects.

#### Experiment 1C

To examine interactions between high- and low-level features, in Experiment 1C memory displays consisted of one face and either one or three Gabor gratings. Depending on which item was probed, participants either had to reproduce the emotional intensity of the face or the orientation of a grating. Facial expressions were chosen randomly from the five emotions as in Experiment 1A. Timings and other experimental variables were identical to the previous experiments.

### Analysis

For face stimuli, we assessed recall bias and variability based on the mean and standard deviation of adjustment errors, defined as the deviation in emotional intensity between target and response. Performance for orientation stimuli was assessed in the same way, based on the angular deviation between target and response, and by using the corresponding circular statistics (Fisher, 1995). Effects of experimental parameters were assessed with standard (null hypothesis) and Bayesian *t* tests and ANOVAs using JASP (Wagenmakers et al., 2018). Bayes factors (BF) are reported in relation to test (BF_10_) or null hypotheses (BF_01_). In two-way ANOVAs, BFs are reported in relation to the model containing the other main effect. Greenhouse-Geisser corrected *p*-values for ANOVAs are reported when the assumption of sphericity was violated; no equivalent correction is available for Bayes factors, to our knowledge, so we do not report BFs in these cases.

### Modeling

To explore the data from the orientation reproduction tasks, we first fit the previously published neural resource model (Bays, 2014) to participant's responses. In this model, based on principles of population coding (Pouget, Dayan, & Zemel, 2000; Pouget, Dayan, & Zemel, 2003), memory stimuli are first encoded in the noisy firing of a population of feature-selective neurons, then reconstructed based on maximum likelihood decoding of the spiking activity. Total activity of the population is held constant (normalized) across changes in set size, with the consequence that the activity associated with any individual item is inversely proportional to set size. The model has two free parameters, the tuning width and gain constant, which determine the width of the tuning functions and the total activity of the population, respectively. Full details of the model can be found in Bays (2014).

We fit the model to data using a grid search, in which the model predictions were first calculated for 60 logarithmically spaced gain and tuning width values between 10^0^ and 10^2^, and then maximum likelihood parameters were obtained for each participant separately. The neural resource model can be fit more precisely using nonlinear optimization (code available at bayslab.com/code/JN14), and this method indeed produced very similar results; however, we used grid search here to facilitate comparison with models described below for which this method is not available.

The existing implementation of the neural resource model cannot be applied directly to errors in the facial expression tasks, because of a difference in the stimulus space. While the set of all possible orientation responses defined a continuous circular space, the set of possible expression intensities defined a one-dimensional Euclidean space bounded at 0% and 100%. Circular stimulus spaces are desirable because they have rotational symmetry: this means a single probability distribution can in theory be rotated around the circle to capture the responses for any target value (in reality this is not precisely true [e.g., Taylor & Bays, 2018], but it is a convenient simplification made by most current models of working memory). A bounded space does not have this property, so for example, a distribution that captures responses to a target at 50% is likely to predict unobtainable, negative intensity responses when translated to a target at 10%.

Our solution was to modify the existing model to incorporate a Bayesian prior distribution into the process of response generation. The existing model generates responses by choosing the stimulus value with maximum likelihood given the simulated spiking activity. This is identical to choosing the maximum of the posterior distribution given a uniform prior distribution. However, for even slightly nonuniform priors, our preliminary investigations found that this procedure tended to produce many responses at precisely the maximum of the prior, a behavior that is not observed in participants' responses. This could be resolved by sampling from the posterior distribution instead of maximizing it. Generating responses by posterior sampling is a method used previously in a model of working memory closely related to the neural resource model (Matthey, Bays, & Dayan, 2015), and it has been argued that it provides a close match to the inference process underlying perceptual decisions (Vul, Goodman, Griffiths, & Tenenbaum, 2014).

To assess the impact of switching from maximum likelihood to posterior sampling in the model, we fit the orientation data again with the posterior sampling modification and a uniform prior. Model predictions were calculated for 50 logarithmically spaced gain values between 10^−1^ and 10^3^ and for 20 logarithmically spaced tuning width values between 10^−1^ and 10^0^ based on simulating a population of 10^3^ neurons. For each possible target value in the parameter space, 10^4^ simulated responses were obtained and a histogram estimate of the distribution calculated based on 40 equally spaced bins. Fits of the maximum likelihood and posterior sampling models were compared using the Akaike Information Criterion (AIC).

Next we fit the posterior sampling model to data from the facial expressions tasks. The only modification required was to change the tuning functions to Gaussian from von Mises (the circular equivalent of the Gaussian). Preferred intensities were evenly spaced in the range 0% to 100%. We set the prior to be uniform over the range 0% to 100% (we also experimented with nonuniform priors but did not find any that substantially improved fit). Model predictions were calculated for 50 logarithmically spaced gain values between 10^−1^ and 10^3^ and for 50 logarithmically spaced tuning width values between 10^−2^ and 10^1^ based on a population of 10^3^ neurons. For each possible target value in the parameter space, 10^5^ simulated responses were generated, and a histogram estimate obtained based on 20 equally spaced bins.

## Results

### Recall of facial expressions

**Figure 2:**
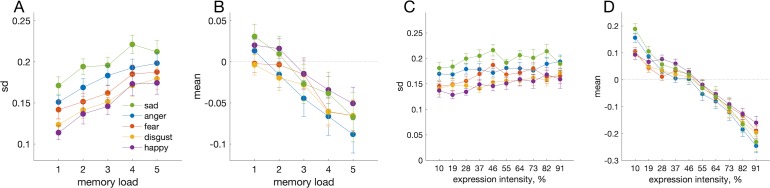
Recall performance for facial expressions. (A) Standard deviation of errors as a function of set size for five different emotional expressions (colored circles). (B) Bias of errors as a function of set size for different emotional expressions. The effect of expression intensity on standard deviation (C) and bias (D) of recall errors.

We measured recall errors for five different types of facial expression. As the memory load (set size) was increased from one to five faces, the variability of errors increased for every expression, *F*(4, 32) = 17.590, *p* < 0.001; BF_10_ = 4.16 × 10^23^ (Figure 2A). There were also significant differences in variability between emotional expressions, *F*(4, 32) = 24.158, *p* < 0.001; BF_10_ = 1.27 × 10^22^: happy and disgusted expressions were remembered most precisely and sad expressions least precisely. No significant interaction between memory load and expression was found, *F*(16, 128) = 0.796, *p* = 0.688; BF_01_ = 75.49.

Set size had small but significant effects on the mean error, *F*(4, 32) = 16.294, *p* < 0.001; BF_10_ = 7.96 × 10^22^: at higher memory loads responses were overall biased towards neutral expressions (Figure 2B). This bias was independent of expression, *F*(4, 32) = 2.852, *p* = 0.102 (BF not calculated due to violation of sphericity) and there was no interaction between set size and expression, *F*(16, 128) = 1.448, *p* = 0.130; BF_01_ = 42.47. While recall variability, measured by the standard deviation of errors, was approximately independent of target intensity (Figure 2C), the bias varied strongly with intensity, *F*(9, 72) = 92.342, *p* < 0.001; BF_10_ = 6.20 × 10^153^ (Figure 2D). This was expected due to the bounded stimulus space: because responses less than 0% or greater than 100% were not possible, responses to targets close to these extremes tend to be biased inwards.

The testing took several hours for each participant and was done over multiple days, which might induce practice effects. With memory loads of two, *t*(7) = 0.247, *p* = 0.812, BF_01_ = 1.93; three, *t*(7) = 1.013, *p* = 0.345, BF_01_ = 1.46; and four, *t*(7) = 0.923, *p* = 0.38, BF_01_ = 1.53, items, there were no statistically significant practice effects (i.e., differences) between the two subgroups of participants that were tested on the set sizes in different order. Only with a memory load of five items, precision was better for the subgroup of participants that had had more practice on the task, *t*(7) = 3.152, *p* = 0.016; BF_10_ = 3.80. When every participant repeated the set size one condition, similar precision to the first measurement was found, *t*(8) = 1.654, *p* = 0.137, BF_01_ = 1.14. A significant practice effect was observed in the time taken to adjust the probe. Over the course of the experiment, the average duration of adjustment gradually decreased from 3.42 to 2.81 s, *F*(4, 32) = 4.971, *p* = 0.003; BF_10_ = 3.02 × 10^11^, with no significant differences between expressions, *F*(4, 32) = 2.592, *p* = 0.135 (BF not calculated due to violation of sphericity), and no significant interaction, *F*(16, 128) = 1.286, *p* = 0.216; BF_01_ = 116.47. When participants repeated the one item condition, the mean adjustment duration remained lower, only 2.50 s. Overall, the practice effects on precision were extremely modest and are not considered further.

To ensure that participants had enough time to encode all the faces in memory, the duration of the memory array was increased in proportion to the number of faces shown (array duration was 1 s/face). To assess the effect of overt attention on performance we calculated the gaze dwell time for each item in a display (i.e., the total time spent fixating that item while the array was present. The mean dwell time for set sizes of one to five was 606 ms, 718, 744, 700, and 725 ms, respectively. Minor correlations between the dwell times on target and non-target faces and memory precision were found. On a trial-by-trial basis, the absolute adjustment error correlated negatively with target dwell time (*r* = −0.0348, *p* < 0.001) and positively with non-target dwell time (*r* = 0.0229, *p* < 0.01), when correlation between the target and non-target dwell time was taken into account (i.e., a partial correlation was calculated). The correlations were in the expected direction (more attention to the target leading to greater recall precision) but very weak.

### Neural resource model

**Figure 3:**
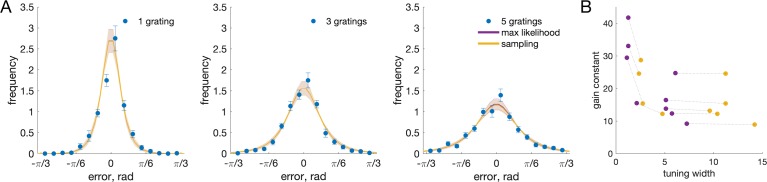
Recall performance for oriented gratings. (A) Mean distributions of response error for orientation recall, for set sizes 1, 3, and 5. The ML decoding (purple curves) and posterior sampling (yellow curves) versions of the neural resource models produced almost identical fits to the data. (B) Best fitting model parameters for each participant.

In order to fit the facial expression data, due to the bounded non-circular space of stimuli used, we had to make some modifications to the neural resource model as previously implemented (Bays, 2014). Specifically, we changed the decoding method from maximum likelihood to posterior sampling (see Methods for details). Before examining the facial expression data, we tested the impact of this modification on fits to orientation recall data, for which both decoding methods could be applied. Figure 3A plots distributions of angular error for set sizes 1, 3, and 5 along with fits of each version of the model. Consistent with previous findings, the neural resource model successfully reproduced both the increase in variability (distribution width) with set size and also the specific shape of the error distributions. Critically, the posterior sampling modification (purple curves) provided fits that were almost indistinguishable from the unmodified model (yellow curves), as confirmed by AIC differences between the two models of −0.2 ± 0.3 (*M* ± *SE*; negative values indicate better fit for unmodified model, *t*(8) = 0.626, *p* = 0.549; BF_01_ = 2.64. The modification did change the parameter values at which these maximum likelihood fits were obtained (Figure 3B), but best fitting parameters were very highly correlated across the two models (gain constant: *r* = 0.89, *p* < 0.01; tuning width: *r* = 0.99, *p* < 0.001).

**Figure 4:**
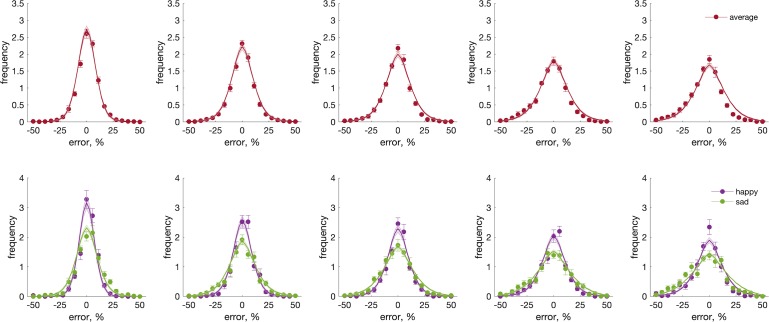
Memory error distributions (symbols) for faces and fits of the population coding model (lines). Error distributions averaged over expression (top row) are shown as well as for happy (bottom row, purple symbols and lines) and sad (bottom row, green symbols and lines) expressions separately, as a function of memory load from one to five faces (plots from left to right). In all plots, data is averaged over participants and target intensities. Data and code for plotting individual error distributions and fits are available at https://osf.io/v79h6/.

**Figure 5:**
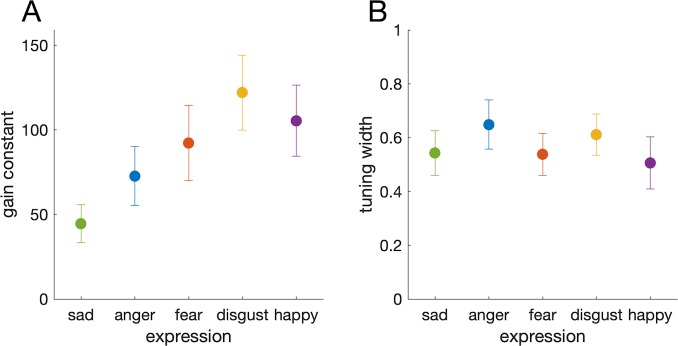
Population coding model parameters for different facial expressions. (A) Gain constants differed between expressions. (B) Tuning widths across expressions were roughly constant.

Having established that the posterior sampling method was an appropriately minor amendment to the existing model, we proceeded to fit this modified model to data from the facial expression task. Figure 4 shows memory error distributions averaged over all expressions (Figure 4, top row) and separately for happy and sad expressions (most precise and most imprecise; Figure 4, bottom row), with average model fits for each set size. The population coding model captured differences between the expressions as well as the effect of memory load. The best fitting gain constants (Figure 5A), *F*(4, 32) = 5.998, *p* = 0.010; BF_10_ = 34.79, were significantly different for different expressions, but tuning widths (Figure 5B), *F*(4, 32) = 0.698, *p* = 0.599; BF_01_ = 4.88, were not. Thus, normalization in the model accounted for the effect of memory load, and different gain constants explained the differences in precision between expressions.

**Figure 6:**
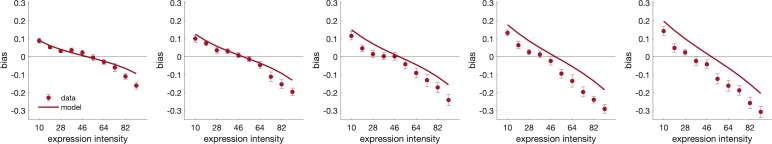
Bias of the error distributions for different set sizes (one to five faces, plots from left to right). Different expressions averaged.

In the behavioral data, a strong bias towards the center of the response space (50%; Figure 2D) was coupled with a smaller bias towards the neutral expression (0%) at larger set sizes (Figure 2B). Interestingly, the population-coding model captured the bias towards the center of the space, and the increase in this bias with set size (Figure 6; left to right), without explicit modeling, simply due to the effect of increasing variability in the bounded stimulus space. At higher set sizes, the bias towards the neutral expression, which was not captured by the model, can be seen as a consistent deviation between the model bias, symmetrical around the mean expression, and the bias in the data (Figure 6). The bias is also visible in the model fits for average expression at set size 5 (Figure 4, top row, rightmost plot). We experimented with reproducing this bias using non-uniform priors, but were unable to markedly reduce the difference in bias between the data and model.

### Competition between faces and orientations in WM

**Figure 7:**
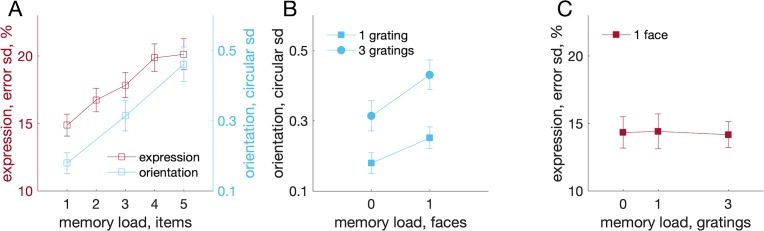
Recall variability for facial expressions and grating orientations. (A) Effect of set size on recall of expressions (red; Euclidean SD, on left y-axis) and orientations (blue; circular SD, on right y-axis), tested separately. (B) Orientation recall for memory arrays consisting of one or three gratings plus zero or one face. Storing a facial expression increases orientation recall variability. (C) Recall of facial expressions for memory arrays consisting of one face plus zero, one, or three gratings. Storing orientations does not affect facial expression recall variability. Note SD values are on different scales for faces and orientations and not directly comparable.

Recall variability for facial expression (mean across emotions) in arrays with differing numbers of faces (Experiment 1A) is shown in Figure 7A as red squares. Blue squares indicate variability of recall for oriented gratings as a function of the total number of gratings in the display (Experiment 1B). Variability increased significantly with set size in both cases (faces: *F*(4, 32) = 21.3, *p* < 0.001; BF_10_ = 7.11 × 10^5^; gratings: *F*(2, 16) = 35.3, *p* < 0.001; BF_10_ = 2.98 × 10^4^.

In the final experiment (Experiment 1C), memory arrays could include both grating orientations and facial expressions, and any of the items could be probed. Figure 7B shows the effect on orientation recall of increasing the number of faces or gratings in the memory array. Variability increased significantly with the number of gratings in the display, one versus three: *F*(1, 16) = 58.09, *p* < 0.001; BF_10_ = 3.52 × 10^4^, and also with the number of faces in the array, zero versus one: *F*(1, 16) = 4.48, *p* = 0.05; BF_10_ = 1.78. The Gratings × Faces interaction was not significant, *F*(1, 16) = 1.20, *p* = 0.289; BF_01_ = 1.83.

Post hoc tests showed that adding a face increased recall variability for arrays of one grating, *t*(16) = 1.78, *p* = 0.047; BF_10_ = 2.14, and three gratings, *t*(16) = 2.07, *p* = 0.028; BF_10_ = 3.06. While the Bayes factors associated with these post hoc tests only indicate modest evidence individually for an effect, the fact that two independent tests (from trials with one or three gratings) both show the same result strongly supports the conclusion that adding a face impairs grating recall.

Figure 7C shows the effect on recall of facial expression of adding gratings to the array. In contrast to the results above, adding one, *t*(8) = 0.122, *p* = 0.906; BF_01_ = 3.09, or three gratings, *t*(8) = 0.334, *p* = 0.747; BF_01_ = 2.96, to the memory array had no significant effect on variability of recall for facial expression (mean over emotions). Here, the two Bayes factors indicate consistent independent evidence *against* an effect of adding gratings on face recall.

## Discussion

We measured visual working memory precision for different emotional expressions of faces, as well as for orientation of Gabor gratings. We found that recall precision for facial expression, like orientation, declined monotonically as the set size was increased from one to five. The decline in memory precision for primary visual features has been explained in terms of a limited memory resource that must be shared out between objects in the environment (Bays et al., 2009; Bays & Husain, 2008; Ma, Husain, & Bays, 2014; van den Berg, Awh, & Ma, 2014; van den Berg et al., 2012; Zhang & Luck, 2008). The present results suggest that working memory for high-level features such as facial expression is similarly resource-limited.

Our results are consistent with those of Lorenc et al. (2014), who tested recall of facial identity using a circular space of faces morphed between different ages and genders. They similarly found that recall variability increased with set size, and additionally that recall was more precise for upright than inverted faces. We did not find better memory performance for angry than happy expressions, as previously reported on an identity memory task (Jackson et al., 2014). In fact, in our experiments happy expressions were the most precisely remembered, in accordance with the advantages for happy expressions previously demonstrated for perceptual identification and recognition (Leppanen & Hietanen, 2004; Svard et al., 2012).

Recent work has related working memory resource to a fixed limit on spiking activity in neural populations encoding visual information (Bays, 2014; Schneegans & Bays, 2017, 2018; Taylor & Bays, 2018). This neural resource model has been shown to accurately reproduce the distributions of recall error and effects of set size on memory for simple visual features, but has not previously been applied to higher-level visual objects. With minor modifications to account for the non-circular, bounded space of expression intensity, we found that this model provided an excellent account of errors in recall of facial expression.

Given the rather arbitrary scaling of the response space for different emotions, we were surprised to find that the differences in precision between emotions were best captured by changes in population gain rather than tuning width. This difference in modeled activity level might reflect differences in the attentional salience of the various emotional expressions. Future work could test this hypothesis, for example, by attempting to match the emotional stimuli based on their reported salience, or by measuring salience of the stimuli indirectly through skin conductance (Alpers, Adolph, & Pauli, 2011), or visual search performance (Calvo & Nummenmaa, 2008). In a separate study examining perceptual identification and discrimination thresholds for different facial expressions, we have found similar rank ordering of expression; that is, sadness is most difficult and happiness is easiest to identify and discriminate. This could indicate a low-level perceptual basis for the differences we observed in recall, although it also does not rule out a salience-based account. We hope to distinguish these possibilities in a future study.

The success of the neural resource model in capturing data could suggest the existence of face-specific neural populations that encode intensity of expressions. In single cell recordings, different subpopulations of face specific cells encode facial identities and expressions (Gothard, Battaglia, Erickson, Spitler, & Amaral, 2007; Hasselmo, Rolls, & Baylis, 1989). fMRI studies also support separate processing of identity and expression (Winston, Henson, Fine-Goulden, & Dolan, 2004). The neurons encoding expressions have been found in amygdala and in the superior temporal sulcus. To our knowledge, no evidence for intensity-tuned neural populations has been found, but this is unsurprising given that these studies have typically compared categorically different faces and objects, facial identities, or expressions, rather than parametrically varying intensity of expression.

In this study we focused on the ability to recall the intensity of facial expressions—as an example of a complex visual property that varies continuously on a one-dimensional scale—rather than the identity of the emotions expressed or the identities of the faces themselves. When recognizing a face, multiple dimensions—identity, expression, and intensity of expression—interact with each other (Galster, Kahana, Wilson, & Sekuler, 2009). Facial expressions, especially happiness, affect face recognition (Liu, Chen, & Ward, 2014), with the best recognition ability observed for neutral faces (Nomi, Rhodes, & Cleary, 2013). Memorizing identities is of course fundamental to social communication, especially on long time scales. However, on shorter time scales, as examined in our study, the ability to store the intensity of expressions could be important for communication also. For example, during a conversation, holding information about visual expression and intensity of expression in working memory likely helps to correctly interpret speech semantics, as well as the mental and emotional states of others.

In previous studies, recall of simple and complex visual objects has been compared using change detection tasks. Alvarez and Cavanagh (2004) found that recall performance declined with object complexity, and suggested that memory capacity depends on the perceptual complexity or information load of the memorized items. A subsequent study found a specific memory advantage for faces over other complex objects, as long as sufficient time was available to encode the stimuli (Curby & Gauthier, 2007). Jiang et al. (2008) compared working memory for line orientations and faces using change detection, and found no overall difference between them when the amount of change was controlled for. However, when memory load was high enough, there was an advantage for faces compared to orientations.

For primary visual features, different feature dimensions (such as orientation, color, and size) appear to recruit largely independent working memory resources (Bays, Wu, & Husain, 2011; Fougnie, Asplund, & Marois, 2010; Shin & Ma, 2017; Wheeler & Treisman, 2002). The evidence for this is little or no cost to recall precision of adding a feature from a different dimension to the memory array compared to adding another feature from the same dimension. However, when our observers were required to memorize orientations and expressions simultaneously, we found an asymmetric effect on recall precision: an additional face stimulus interfered with orientation recall, but additional oriented gratings had no effect on recall of facial expressions. A simple explanation for this is that faces contain both high- and low-level feature information. When faces are perceived and encoded in memory, orientation information as well as face-specific information is stored. Storing this orientation information takes up resources that are also required for storing the orientations of gratings, resulting in poorer recall of the gratings. But adding a grating to a face array does not have any impact on face-specific resources, so recall of facial expressions is unaffected.

This account rests on the assumption that orientation information is automatically encoded from face stimuli, despite being unnecessary for the task (as shown by the absence of a performance cost when orientation resources are distributed to additional gratings). The extent to which memory resources can be selectively allocated to different feature dimensions of a single object has been the subject of several previous studies. Marshall and Bays (2013) found that observers showed identical recall performance when instructed to selectively store only the colors of one set of colored bars and only the orientations of another set, as when they were asked to store all features of both sets of items. This suggests that memory resources could only be allocated at the object and not individual-feature level. In a subsequent experiment, simply comparing the colors of two oriented bars displayed together was sufficient to disrupt recall of other orientations held in memory, to the same extent as if participants were instructed to memorize the orientations of the bars. When the stimuli to be compared did not contain orientation information they did not disrupt memory for orientation. This suggests that merely attending to one feature of an item is sufficient to encode all its features in memory.

While Marshall and Bays (2013) showed that the distribution of WM resources to an attended object is automatic, two studies that used “one-shot” experimental designs found that unattended feature dimensions were poorly recalled (Chen & Wyble, 2015; Shin & Ma, 2016). These studies tested performance on “surprise” trials in which participants were unexpectedly asked to report a feature they had not been instructed to memorize, and found that recall was much less accurate than on trials where the expected feature was requested. One possible synthesis of these results would suggest that irrelevant features are encoded (taking up resources) but not subsequently maintained in memory. Alternatively, the demand to suddenly and unexpectedly perform a task on which one has no previous experience may be sufficient to disrupt memory maintenance and so explain poor performance on surprise tests.

Face specific neurons have been found both in monkey and human single cell recordings (Quiroga, Reddy, Kreiman, Koch, & Fried, 2005; Wang et al., 2014). The processing of faces is distributed over several brain areas (Barraclough & Perrett, 2011), in which different populations of neurons encode facial identities, viewpoints, and emotional expressions (Gothard et al., 2007; Hasselmo et al., 1989). Decoding of fMRI activity patterns during working memory maintenance suggests memory representations can be found in visual (Christophel & Haynes, 2014; Harrison & Tong, 2009; Serences, Ester, Vogel, & Awh, 2009), parietal and frontal areas depending on the memorized visual stimuli, memory task (Lee, Kravitz, & Baker, 2013), and the level of abstractness of representation (Christophel, Klink, Spitzer, Roelfsema, & Haynes, 2017). Facial identity can be decoded from the activity patterns in temporal (Kriegeskorte, Formisano, Sorger, & Goebel, 2007; Natu et al., 2010) and frontal (Guntupalli, Wheeler, & Gobbini, 2017) brain areas, and facial expression in occipital and temporal areas (Liang et al., 2017). Memorized faces can also be reconstructed on the basis of activation patterns in the angular gyrus of the parietal cortex (Lee & Kuhl, 2016). Taken together, one interpretation of these studies is of a processing hierarchy in which simple visual features can be decoded from primary visual cortex but decoding more complex stimuli requires signals from association cortex. Our results showing asymmetric competition for memory resources for gratings and faces are consistent with this view; neural populations in primary visual cortex could encode orientation information (from both faces and gratings) while populations in face-specific regions higher in the processing hierarchy encode facial expression.
